# Evolution of Pulmonary Embolism Response Teams in the United States: A Review of the Literature

**DOI:** 10.3390/jcm13133984

**Published:** 2024-07-08

**Authors:** Vidish Pandya, Akhil Avunoori Chandra, Andrea Scotti, Manaf Assafin, Aldo L. Schenone, Azeem Latib, Leandro Slipczuk, Asma Khaliq

**Affiliations:** Division of Cardiology, Montefiore Health System, Albert Einstein College of Medicine, 111 E 210TH ST, Bronx, NY 10467, USA; vipandya@montefiore.org (V.P.);

**Keywords:** PERT, PE, thrombectomy, thrombolysis, anticoagulation, embolectomy multidisciplinary

## Abstract

Pulmonary embolism (PE) is a significant cause of cardiovascular mortality, with varying presentations and management challenges. Traditional treatment approaches often differ, particularly for submassive/intermediate-risk PEs, because of the lack of clear guidelines and comparative data on treatment efficacy. The introduction of pulmonary embolism response teams (PERTs) aims to standardize and improve outcomes in acute PE management through multidisciplinary collaboration. This review examines the conception, evolution, and operational mechanisms of PERTs while providing a critical analysis of their implementation and efficacy using retrospective trials and recent randomized trials. The study also explores the integration of advanced therapeutic devices and treatment protocols facilitated by PERTs. PERT programs have significantly influenced the management of both massive and submassive PEs, with notable improvements in clinical outcomes such as decreased mortality and reduced length of hospital stay. The utilization of advanced therapies, including catheter-directed thrombolysis and mechanical thrombectomy, has increased under PERT guidance. Evidence from various studies, including those from the National PERT Consortium, underscores the benefits of these multidisciplinary teams in managing complex PE cases, despite some studies showing no significant difference in mortality. PERT programs have demonstrated potentials to reduce morbidity and mortality, streamlining the use of healthcare resources and fostering a model of sustainable practice across medical centers. PERT program implementation appears to have improved PE treatment protocols and innovated advanced therapy options, which will be further refined as they are employed in clinical practice. The continued expansion of the capabilities of PERTs and the forthcoming results from ongoing randomized trials are expected to further define and optimize management protocols for acute PEs.

## 1. Introduction

Pulmonary embolism (PE) affects between 350,000 and 900,000 individuals annually in the United States. It is the third leading cause of cardiovascular death, with a 30-day mortality rate of 10% and a 1-year mortality rate of up to 20% [1,2,3,4,5]. In clinical settings, PEs are classified using the PE Severity Index (PESI) into three categories—low risk, submassive/intermediate risk, and massive/high risk—based on clinical features such as hypotension, need for vasopressor support, and right-ventricular strain or dysfunction [6,7]. The Wells and Geneva scoring systems, echocardiograms/bedside point-of-care ultrasounds, spiral computed tomography angiography (CTA) scans, and PE Rule out Criteria (PERCs) are also used when a diagnosis is not immediately clear. Once a PE diagnosis is confirmed, the universally accepted initial treatment is systemic anticoagulation (unfractionated heparin, argatroban, bivalirudin, etc.). More aggressive interventions, including systemic thrombolysis, catheter-directed therapy (lytic or thrombectomy), surgical embolectomy, and mechanical circulatory support, are reserved for severe cases according to established guidelines [8] [Table 1 and Table 2].

While treatment protocols for massive/high-risk and low-risk PEs are generally standardized, the approach to managing submassive/intermediate-risk PEs is less clearly defined [Table 3]. This is further complicated by discrepancies and variations among guidelines from various societies, including the American Heart Association (AHA) [9], American College of Chest Physicians (ACCP) [10], American Society of Hematology (ASH) [11], and European Society of Cardiology (ESC) [12]. 

This variability in management, along with the urgent need to devise a treatment plan, forces clinical teams to rapidly evaluate and decide on the best course of action for these complex cases, often without a clearly superior treatment option. Variations in management are further influenced by the expertise and resources available at local institutions. Consequently, physicians must often weigh the risks of rapidly initiating potential therapies—such as bleeding, procedural complications, and mortality—against the dangers of treatment delays [13].

The past decade has seen the emergence of Pulmonary Embolism Response Teams (PERTs) in reaction to the abovementioned challenges, with a particular focus on addressing intermediate and high-risk PEs, a class IIa recommendation by the European Society of Cardiology [12]. These multidisciplinary units are designed to rapidly and comprehensively manage acute pulmonary embolisms (PEs). Comprising specialists from various fields, including emergency medicine, pulmonology, critical care, radiology, interventional radiology, interventional cardiology, cardiothoracic surgery, and hematology, PERTs guide treatment decisions based on the patient severity, ranging from anticoagulation to fibrinolysis, catheter-directed therapies, or surgical embolectomy. We conducted a review of the current trends in the management and treatment of PEs, focusing on data related to PERTs. This review aims to examine the conception, evolution, implementation, and effectiveness of PERTs, as well as discuss the current and future state of these programs based on available literature. 

## 2. Conception and Evolution of PERT in the United States

The first Pulmonary Embolism Response Team in the United States was established in 2012 at Massachusetts General Hospital, affiliated with Harvard Medical School. This multidisciplinary team, composed of specialists from cardiovascular medicine, cardiothoracic surgery, emergency medicine, pulmonary and critical cares, vascular medicine, and radiology, was designed to provide real-time, consensus-driven management recommendations for patients with massive and submassive PEs.

Initially, a PERT was activated via telephone for a confirmed or suspected severe PE. A designated physician member would evaluate the case, considering patient hemodynamics, clinical characteristics, and available imaging to determine the necessity of convening a full team meeting. This physician also had the authority to mobilize essential resources, such as the cardiac catheterization lab or operating room and associated staff, depending on the decided course of treatment. 

The benefits of PERTs were demonstrated through a case series of two complex situations in which rapid, expert consensus was crucial because of the lack of clear evidence on available treatment options [14,15]. In these cases, the PERT facilitated the inclusion of additional specialties, such as OBGYNs, maternal–fetal medicine, vascular surgery, and structural heart, to formulate optimal treatment plans. One remarkable instance involved coordinating expedited care for a patient presenting at an external hospital, showcasing a PERT’s capability of extending its expert management to more complex, time-sensitive cases, even beyond its home institution.

This pioneering initiative laid the groundwork for the formation of the National PERT Consortium in 2015, when physicians–experts met in Boston, Massachusetts, to create a collaborative environment to guide treatment by PERTs and medical institutions around the world [8]. The mission of this international nonprofit organization was “to facilitate the exchange of ideas and information related to the care of patients with PE, and to advance the science of PE care by performing research, developing advanced treatment protocols, and educating clinicians and community members”. At that meeting, the National PERT Consortium released an executive summary to guide the diagnosis and treatment (including advanced therapies) for patients presenting with an acute PE. 

Physician attendees at the 2015 National PERT Consortium were also surveyed to collect responses regarding treatment of choice for various clinical scenarios to guide clinical practice. For example, a patient with a high-risk PE, low bleeding risk, right-ventricular dysfunction, and a right atrial clot was deemed to be an acute submassive, high-risk PE in line for surgical embolectomy by 77 respondents (88% of survey respondents) [8]. However, this survey did note that respondents with access to PERT programs were more likely to suggest interventional and surgical therapies to treat patients. Since then, research revolving around PERT programs in the United States has advanced greatly with numerous retrospective trials and active randomized control trials.

To follow-up on the evolution of PERT programs across the nation, Barnes et al. surveyed active National PERT Consortium members on their respective institutions. An online questionnaire administered to 31 institutions (of which 19 had PERT programs) noted that most PERTs involved 3–5 different specialties which worked together primarily via telecommunication (12/18, 67%). Nearly 90% of these centers offered 24/7 advanced therapies albeit with variations regarding individual steering committee members [16]. Interestingly, many programs (nearly 50%) offered dedicated “PERT clinics” for patients to follow-up in after hospitalization. 

The first reports regarding PERTs were published around 2018 by Rosovsky et al., examining a 10-year analysis regarding outcomes at a tertiary-care institution [17]. In their study, Rosovsky et al. compared the pre- and postinterventions of a PERT program against two prior studies with a control cohort of PE patients who were seen in their institution’s emergency department (the prospective safe pulmonary embolism emergency department discharge (SPEED-D) study and the retrospective, SPEED-D(R) study). Their study identified 212 patients before a PERT intervention and 228 patients after a PERT intervention who met the inclusion criteria, and they noted that the proportion of patients undergoing catheter-directed therapy (10 [1%] vs. 31 [14%], unadjusted *p* < 0.0001) and any advanced therapy (19 [9%] vs. 44 [19%], unadjusted *p* = 0.002) was higher in the post-PERT group. Thus, noting that PERT increased the use of catheter-directed interventions and additional advanced therapies with no associated changes in bleeding or mortality for patients with massive and submassive PE. 

The evolution of PERT was further accelerated by the COVID-19 pandemic because of the pathophysiologic and thrombogenic nature of COVID-19 infection and resultant downstream effects including sepsis and coagulopathy [18]. The position of the aforementioned paper recommended that hemodynamically stable patients presenting with COVID-19 infection should be managed with anticoagulation alone while unstable patients or patients pending imminent deterioration should be considered for thrombolysis. Catheter-based thrombolysis and/or surgical embolectomy was cautiously recommended when no other alternatives were feasible because of concerns about viral contamination of the operating rooms and catheterization labs. In one prior report from an urban tertiary-care center from a 2-month period in 2020, there was a statistically significant decrease in catheter-directed therapies (5.5% vs. 23.1%, *p* = 0.02) during the COVID-19 pandemic with a clinically significant trend toward systemic fibrinolytic therapy during the same time period (13.5% vs. 3.9%, *p* = 0.3) [19]. Nevertheless, PERT program utilization was nearly three-fold higher during the study time.

Since the pandemic, PERTs have become more commonplace in both university and community medical centers [15,20]. Studies have investigated the utilization and outcomes of PERTs, with one paper, by Myc et al., noting that the rate of mortality significantly decreased with the adoption of a PERT program without additional increases in hospital-based costs or lengths of hospital stay [21]. Their team further noted a significant decrease in 30-day readmission rates in patients who had been treated through a PERT program (nearly 10%, *p* = 0.047).

To facilitate the treatment of patients at PERT-capable facilities with options for advanced therapies, Rali et al. previously enumerated a flow pathway for non-PERT-capable facilities to create an “Interhospital Transfer of Patients With Acute Pulmonary Embolism”, with specific protocols on how to manage acute PEs with advanced management, such as critical care and vasopressor support [22]. Further, Rosovsky et al. also published a treatment algorithm on how to escalate from anticoagulation to advanced therapies in cases of acute PE through the use of clinical guidelines and case-based approaches [23]. 

## 3. Outcomes of PERT

PERT programs include individuals from various specialties who all typically participate in the initial and long-term management of PEs. These specialties include pulmonology, hematology, cardiology (invasive and noninvasive), radiology (diagnostic and interventional), emergency medicine, and cardiothoracic surgery. This team collaborates to assess and manage patients with an acute PE, particularly those who are hemodynamically unstable or have a high risk of complications. The response is coordinated through a centralized communication system, often activated by a single-caller paging system to create a treatment plan by pooling resources. In PERT programs, the severity of an embolism and patient-specific factors are evaluated to tailor therapy. This is conducted through a standardized protocol that streamlines care and reduces the variability in treatments with the goal of improving outcomes [15,24]. 

A main objective of PERT implementation is to diagnose and effectively triage high-risk patients with a massive PE before clinical deterioration to obstructive shock and/or cardiac arrest. Bedside point-of-care ultrasounds or echocardiograms may be used to immediately triage patients, and this has previously been reported to have a sensitivity of 74%, specificity of 95%, positive predictive value of 95%, and negative predictive value of 75% in diagnosing pulmonary embolisms [25]. CTA should be pursued if point-of-care ultrasounds or echocardiograms are equivocal or clinical suspicion of PE is high. In such patients, systemic thrombolysis through peripheral access is the treatment of choice in the absence of any absolute contraindications (Table 1 and Table 2). While performing this triage, PERT members also evaluate patients for candidacy for veno-arterial extracorporeal membrane oxygenation (VA-ECMO), which may function as a bridge to additional therapies such as catheter-based interventions and surgical embolectomy [26]. It should be noted that systemic thrombolysis does not preclude future cannulation to institute VA-ECMO. Surgical embolectomy is the first-line treatment of choice for high-risk patients with massive PEs presenting with a clot in transit or a right-ventricular thrombus. The SPEAR working group evaluated 214 patients with an acute PE treated with surgical embolectomy and noted a mortality rate of 11.7%. They further noted that mortality was highest among those patients who experienced perioperative cardiac arrest [27]. A recent meta-analysis by Maqsood et al. pooled 492 patients with a clot in transit and a PE and noted that weighted mortality for anticoagulation alone was 35% (95% CI 21–49%), surgical thrombectomy was 31% (95% CI 16–47%), catheter-based thrombectomy was 20% (95% CI 6–34%), and systemic thrombolytic therapy was 12% (95% CI 5–19%) [28] [Table 2].

**Table 1:** Pulmonary embolism risk stratification.

Finding	Low Risk	Intermediate Risk (Submassive)	High Risk (Massive)
Hemodynamic Instability ^a^	−	−	+
Pulmonary Embolism Severity Index (PESI)	Low-risk PESI/sPESI	PESI Classes III–IV sPESI ≥ 1	Stratification not required in shock or instability
Cardiac Biomarker (Troponin, BNP)	−	+	+
Right Ventricular (RV) Strain	−	+/− ^b, c^	+ ^b, c^
“Red Flag” Clinical Findings	−	−	Syncope Clot in transit Cardiac Arrest
^a^ Defined as systolic blood pressure <90 mmHg for ≥15 min or a decrease in systolic blood pressure of ≥40 mmHg or vasopressor use ^b^ RV strain on TTE: - Dilatation of RV (basal diameter > 4.2 cm, mid-cavity diameter > 3.5 cm, and length > 8.2 cm); - RV/LV end-diastolic basal-diameter ratio > 1; - Intraventricular septal flattening; - Paradoxical septal motion. ^c^ RV strain on CT: - Abnormal positioning of intraventricular septum; (normal = septal bowing toward RV; abnormal = septal bowing toward LV); - Paradoxical interventricular septal bowing; - RV enlargement (increased RV/LV ratio > 0.9); - Pulmonary trunk enlargement; - RV failure (inferior vena cava reflux, dilatated azygous, or hepatic veins).			

**Table 2:** Recent studies on pulmonary embolism response teams.

Author	Year	Study Type	Control Group?	Study Population	Sample Size	Main Outcomes
Mahar et al., 2018 [29]	2018	Retrospective	No	PERT Activations	134	35% received advanced therapies (12% catheter-directed thrombolysis (CDT)); decrease in 30-day mortality
Sista et al., 2018 [30]	2018	Retrospective	No	Acute PEs	124	CDT administered to 25 patients, systemic thrombolysis (ST) to 6, and anticoagulation alone (AC) to 54
Chaudhury et al., 2019 [31]	2019	Retrospective	Yes—Historical control group	Acute PEs	pre-PERT = 343 post-PERT = 426	Lower rates of bleeding (17% pre vs. 8.3% post), shorter times to therapeutic AC (16.3 h pre vs. 12.6 h post), decrease use of IVC filters (22.2 vs. 16.4%), and decreased 30-day/inpatient mortality
Khaing et al., 2019 [32]	2019	Retrospective	No	PERT Activations	52	16/52 patients underwent CDT with lower but not statistically significant intensive-care-unit (ICU) length of stay (3 vs. 4 days) and hospital LOS (4 vs. 5 days)
Rosovsky et al., 2019 [17]	2019	Retrospective	Yes—Historical control group	Acute PEs	pre-PERT = 212 post-PERT = 228	Increases in advanced therapies (9% pre vs. 19% post) and catheter-directed therapies (1% pre vs. 14% post). There were no differences in bleeding and mortality
Schultz et al., 2019 [33]	2019	Retrospective	No	PERT Activations	475	The number of activations at each institution ranged from 3 to 13 activations/month/1000 beds, with the majority originating from the emergency department (281/475; 59.3%). AC alone was the most common therapy (289/416 (70%)) in patients with a confirmed PE. The 30-day mortality was 16% (53/338)
Wiske et al., 2019 [34]	2019	Retrospective	No	PERT Activations	201	Most patients were treated without invasive intervention; 91.4% (95% confidence interval [CI], 87.1–95.7%) of patients received anticoagulation alone, 4.5% (95% CI, 0–18.6%) had CDT, and 3.0% (95% CI, 0–16.9%) had systemic administration of tissue plasminogen activator (tPA). There was no difference in the mortality rates of patients who underwent aggressive management compared to anticoagulation alone
Xenos et al., 2019 [35]	2019	Retrospective	Yes—Historical control group	Mortality	pre-PERT = 992 non-PERT = 77	There was no statistically significant difference in the mortality rates between the two groups. The PERT group had significantly shorter ICU stays and overall LOS. No difference was seen in the direct cost between the two groups despite higher use of interventional treatment modalities in the PERT group
Carroll et al., 2020 [36]	2020	Retrospective	Yes—Historical control group	Acute PEs	pre-PERT = 884 post-PERT = 1158	No difference in mortality, increase in catheter-directed therapies (1.3% pre vs. 3.3% post), decrease in systemic thrombolysis (3.8% pre vs. 2.1% post), and IVC filter use (10.7% pre vs.6.9% post)
Melamed et al., 2020 [37]	2020	Retrospective	Yes—Historical control group	Acute PEs	pre-PERT = 317 post-PERT = 411	Increase in advanced therapies (4.7% to 16.1%) and decrease in hospital LOS (4.78 vs. 2.81 days)
Myc et al., 2020 [21]	2020	Retrospective	Yes—Historical control group	Acute PEs	pre-PERT = 237 post-PERT = 120	Decrease in mortality, lower 30-day readmission rates, and increase in the use of advanced therapies in PERT era
Annabathula et al., 2021 [38]	2021	Retrospective	Yes—Historical control group	Acute PEs	pre-PERT = 226 post-PERT = 304	Median decreases in the LOS of 3 days, ICU LOS of 1.5 days, and in-hospital mortality (16.5% pre vs. 9.5% post)
Araszkiewicz et al., 2021 [39]	2021	Retrospective	No	PERT Activations	680	23.3% received advanced therapies
Parikh et al., 2021 [40]	2021	Prospective	No	PERT Activations	307	PERT activated for 22.5% of PEs with increase in advanced therapies (35% vs. 2%) without increase in bleeding complications
Wright et al., 2021 [41]	2021	Observational	Yes—Historical control group	Mortality	pre-PERT = 137 post-PERT = 231	PERT associated with a reduction in mortality through 6 months (14% post-PERT vs. 24% pre-PERT). There was a reduced length of stay following PERT implementation (9.1 vs. 6.5 days, *p* = 0.007)
Ardeshna et al., 2023 [42]	2023	Retrospective	Yes—Historical control group	Acute PEs	pre-PERT = 168 post-PERT = 649	Decrease in advanced therapies (16% pre vs. 7.5% post) and no difference in ICU or hospital LOS and 30 all-cause mortalities

**Table 3:** Recent trials on pulmonary embolism therapies.

a. Catheter-Directed Therapies.					
Trial Name	Author	Study Type	Therapy Studied	Exclusion Criteria	Main Outcomes
ULTIMA	Kucher et al., 2014 [43]	RCT	Ultrasound-assisted catheter-directed thrombolysis (USAT, n = 30) vs. heparin (n = 29)	Age < 18 or >80 years; index PE symptom duration > 14 days; insufficient echocardiographic image quality; known bleeding risk; thrombolytic agents within 4 days; hematologic abnormalities, malignancy, and terminal illness	In the USAT group, the mean RV/LV ratio decreased from 1.28 ± 0.19 at baseline to 0.99 ± 0.17 at 24 h (*p* < 0.001); in the heparin group, mean RV/LV ratios were 1.20 ± 0.14 and 1.17 ± 0.20, respectively (*p* = 0.31).
SEATTLE II	Piazza et al., 2015 [44]	Prospective	Ultrasound-facilitated catheter-directed, and low-dose fibrinolysis, using the EkoSonic Endovascular System (n = 150)	Stroke and intracranial or intraspinal disease within 12 months; major surgery within 7 days; recent active bleeding; hematologic abnormalities; serum creatinine >2 mg/dL; and systolic blood pressure <80 mm Hg despite vasopressor or inotropic support	Mean RV/LV diameter ratio decreased from baseline to 48 h postprocedure (1.55 vs. 1.13; mean difference, −0.42; *p* < 0.0001).
OPTALYSE	Tapson et al., 2018 [45]	RCT	Ultrasound-facilitated catheter-directed thrombolysis (USCDT) for the treatment of acute intermediate-risk (submassive) pulmonary embolisms (n = 101)	Stroke, head trauma, and recent active bleeding from a major organ within 1 month, major surgery within 7 days of screening, systolic blood pressure <90 mm Hg, or use of vasopressors, hematologic abnormalities, and creatinine outside the normal range	Treatment with US CDT using a shorter delivery duration and lower-dose tissue plasminogen activator was associated with improved RV function and reduced clot burden compared with baseline.
FLARE	Tu et al., 2019 [46]	Prospective	Catheter-directed mechanical thrombectomy for intermediate-risk acute pulmonary embolisms (n = 106)	Exclusion criteria included thrombolytic therapy within 30 days of baseline assessment, active cancer, or contraindication to anticoagulation	At 48 h postprocedure, the average RV/LV ratio reduction was 0.38 (25.1%; *p* < 0.0001).
SUNSET-PE	Avgerinos et al., 2021 [47]	RCT	Standard (CDT, n = 41) versus ultrasound-assisted thrombolysis (USAT, n = 40) for submassive pulmonary embolism	Age <18 years, symptoms for >14 days, elevated bleeding risk, participation in any other investigational drug or device study, and life expectancy <90 days	The mean reduction in RV/LV ratio from baseline (1.54 ± 0.30 for USAT and 1.69 ± 0.44 for CDT) to 48 h was 0.37 ± 0.34 in the USAT group and 0.59 ± 0.42 in the CDT group (*p* = 0.01).
EXTRACT-PE	Sista et al., 2021 [48]	Prospective	Indigo aspiration system for treatment of pulmonary embolism (n = 119)	Tissue-type plasminogen activator administration within 14 days of baseline CTA, peak pulmonary artery pressure >70 mm Hg, fraction of inspired oxygen requirement >40% or >6 L/min to keep oxygen saturations >90%, cardiovascular or pulmonary surgery within last 7 days, cancer requiring active chemotherapy, life expectancy <90 days, and major trauma <14 days	Mean RV/LV ratio reduction from baseline to 48 h postprocedure was 0.43 (95% CI: 0.38 to 0.47; *p* < 0.0001).
A Pilot Randomized Trial of Catheter-Directed Thrombolysis or Standard Anticoagulation for Patients with an Intermediate- and High-Risk Acute Pulmonary Embolism	Kroupa et al., 2022 [49]	RCT	CDT (n = 12) vs. standard anticoagulation (n = 11) for patients with an intermediate/high-risk PEs	Clinically significant bleeding, any hemorrhagic stroke, ischemic stroke <6 months ago, recent brain surgery, major surgery 7 days prior, RV/LV ratio <0.7 on TTE, active malignancy, and hematologic abnormalities	An RV/LV ratio decrease of ≥25% (evident on computed tomography angiography) was achieved in 7 of 12 patients in the CDT group vs. 2 of 11 patients in the standard care group (*p* = 0.03). A systolic pulmonary artery pressure decrease of ≥30% or normotension at 24 h after randomization was present in 10 of 12 patients in the CDT group vs. 2 of 11 patients in the standard care group (*p* = 0.001).
CANARY	Sadeghipour et al., 2022 [50]	RCT	Catheter-directed thrombolysis (CDT, n = 48) vs. anticoagulation (n = 46) in patients with acute intermediate/high-risk pulmonary embolisms	Creatinine clearance < 30 mL/min, contraindications to fibrinolytic therapy, concomitant right heart thrombosis, or terminal illness	The median (IQR) 3-month RV/LV ratio was significantly lower with CDT (0.7 [0.6–0.7]) than with anticoagulation (0.8 [0.7–0.9]; *p* = 0.01). An RV/LV ratio greater than 0.9 at 72 h after randomization was observed in fewer patients treated with CDT (13 of 48 [27.0%]) than anticoagulation (24 of 46 [52.1%]; OR, 0.34; 95% CI, 0.14–0.80; *p* = 0.01).
FLAME	Silver et al., 2023 [51]	Prospective	FlowTriever mechanical thrombectomy (FlowTriever arm, n = 53) or with other contemporary therapies (Context arm, n = 61)	Out-of-hospital cardiac arrest with Glasgow Coma Scale score ≤8, witnessed cardiac arrest with ongoing cardiopulmonary resuscitation ≥30 min, and history or current evidence of medical conditions or participation in other clinical studies that would preclude enrollment	The primary endpoint was an in-hospital composite of all-cause mortality, bailout to an alternate thrombus removal strategy, clinical deterioration, and major bleeding. The primary endpoint was reached in 9/53 (17.0%) of patients in the FlowTriever arm (significantly lower than the 32.0% performance goal (*p* < 0.01)) and 39/61 (63.9%) in the Context arm. In-hospital mortality occurred in 1/53 (1.9%) patients in the FlowTriever arm and in 18/61 (29.5%) patients in the Context arm.
FLASH	Toma et al., 2023 [52]	Prospective	Mechanical thrombectomy for intermediate/high-risk PEs (n = 800)	Patients unable to receive AC and those with a life expectancy <30 days	A 7.6 mmHg mean drop in mean pulmonary artery pressure (−23.0%; *p* < 0.0001) and a 0.3 L/min/m^2^ mean increase in cardiac index (18.9%; *p* < 0.0001) in patients with depressed baseline values. At 48 h, RV/LV ratio decreased from 1.23 ± 0.36 to 0.98 ± 0.31 (*p* < 0.0001).
**b. Anticoagulation and Fibrinolytic Therapies**					
**Trial Name**	**Author**	**Study Type**	**Therapy Studied**	**Exclusion Criteria**	**Main Outcomes**
Alteplase Versus Heparin in Acute Pulmonary Embolism: Randomized Trial Assessing Right-Ventricular Function and Pulmonary Perfusion	Goldhaber et al., 1993 [35]	RCT	Recombinant tissue plasminogen activator (rt-PA, n = 46) 100 mg over 2 h followed by intravenous heparin versus heparin alone (n = 55)	Major internal bleeding in the previous 6 months; intracranial or intraspinal disease; operation or biopsy in the preceding 10 days (or open heart surgery within 14 days); blood pressure greater than 200 mm Hg systolic or 110 mm Hg diastolic; severe impairment of hepatic function; pregnancy; active infective endocarditis; hemorrhagic retinopathy; or any concurrent condition considered to limit survival to within one month	Pulmonary perfusion scans were obtained at baseline and 24 h. In 39% of patients administered alteplase (rt-PA) but only 17% of patients administered heparin alone, the right-ventricular wall motion at 24 h improved from baseline and in 2% and 17%, respectively, it worsened (*p* = 0·005). Patients administered rt-PA also had a significant decrease in right-ventricular end-diastolic area over the 24 h after randomization and a significant absolute improvement in pulmonary perfusion (14·6% vs. 1·5%).
Bolus Tenecteplase for Right Ventricle Dysfunction in Hemodynamically Stable Patients with Pulmonary Embolism	TIPES Study Group [53], 2010	RCT	Tenecteplase 30–50 mg plus heparin (n = 23) vs. placebo (n = 28)	Chronic pulmonary hypertension, severe COPD, or hypertension (systolic blood pressure > 180 mm Hg and/or diastolic blood pressure >110 mm Hg), recent bleeding, active peptic ulcer, arterial aneurysm, or arterial/venous malformation	The decrease in the RV/LV ratio at 24 h was 0.31 ± 0.08 in patients randomized to tenecteplase compared to 0.10 ± 0.07 in patients randomized to placebo (*p* = 0.04).
Six-Month Echocardiographic Study in Patients With Submassive Pulmonary Embolism and Right Ventricle Dysfunction: Comparison of Thrombolysis With Heparin	Fasullo et al., 2011 [30]	RCT	Thrombolysis (heparin plus alteplase group, n = 37) on RVD in hemodynamically stable patients with submassive PE vs. placebo (n = 35)	Active internal bleeding, recent intracranial bleeding, ischemic stroke until 2 months, neurosurgery during last month, recent surgery within 10 days, trauma within 15 days, uncontrolled hypertension (SBP >180 mm Hg and diastolic BP >110 mm Hg), hemorrhagic disorder of thrombocytopenia (<100,000), severe impaired hepatic or renal function, pregnancy, and age older than 75 years	Thrombolysis group showed a significant early improvement in RV function compared with heparin group, and this improvement was observed also during the follow-up (180 days).
TOPCOAT	Kline et al., 2014 [39]	RCT	Low-molecular-weight heparin followed by random assignment to either a single weight-based bolus of tenecteplase (n = 40) or Placebo (n = 43)	Contraindications to fibrinolysis or frailty precluding the 6 min walk test (228, 41%), investigator unavailable (144, 26%); clinical care team decided to administer fibrinolytics (139, 25%), creatinine clearance < 30 mL min^−1^ (22, 4%), and other situations precluding follow-up (22, 4%)	83 patients were randomized; 40 to tenecteplase and 43 to placebo. Within 5 days, adverse outcomes occurred in three placebo-treated patients (death in one and intubation in two) and one tenecteplase-treated patient (fatal intracranial hemorrhage). At 90 days, adverse outcomes occurred in 13 unique placebo-treated patients and five unique tenecteplase-treated patients. Thus, 16 (37%) placebo-treated and six (15%) tenecteplase-treated patients had at least one adverse outcome (exact two-sided *p* = 0.017).
PEITHO	Meyer et al., 2014 [27]	RCT	Fibrinolysis (tenecteplase) for intermediate-risk PEs (n = 506) vs. placebo (n = 499)	Hemodynamic decompensation, known bleeding risk, thrombolytic therapy in previous 4 days, inferior vena cava filter or pulmonary thrombectomy in previous 4 days, and known coagulation disorder	Death or hemodynamic decompensation occurred in 13 of 506 patients (2.6%) in the tenecteplase group compared to 28 of 499 (5.6%) in the placebo group (OR, 0.44; 95% CI: 0.23 to 0.87; *p* = 0.02). Between randomization and day 7, a total of 6 patients (1.2%) in the tenecteplase group and 9 (1.8%) in the placebo group died (*p* = 0.42).
MAPPET	Konstantinides et al., 2022 [51]	RCT	Heparin plus 100 mg of alteplase (n = 118) or heparin plus placebo (n = 138) over a period of two hours	Age > 80 years; hemodynamic instability, cardiogenic shock; surgery within the past 7 days, neurologic surgery within the preceding 6 months; gastrointestinal bleeding within the preceding 3 months; inability to tolerate alteplase; diabetic retinopathy; current therapy with an oral anticoagulant; current pregnancy or lactation; and a life expectancy of less than 6 months	The primary endpoint was in-hospital death or clinical deterioration requiring an escalation of treatment. The incidence of the primary endpoint was significantly higher in the heparin-plus-placebo group than in the heparin-plus-alteplase group [34 (24.6%) vs. 13 (11.0%), *p* = 0.006], and the probability of 30-day event-free survival was higher in the heparin-plus-alteplase group (*p* = 0.005).

There are minimal trials comparing outcomes post-PERT versus those pre-PERT with most studies employing observational data from prior studies to create comparative arms. One of the longest PERT study periods to date was conducted by Rosovsky et al., 2018 and was a 10-year analysis of changes in treatment outcomes after establishment of PERT. Their team utilized a pre and postintervention study using an interrupted time series design to compare PE patients pre-PERT (2006–2012, n = 212) vs. post-PERT (2012–2016, n = 228) [17]. Their team found that post-PERT patients were more likely to undergo catheter-directed therapy (1% vs. 14%, *p* ≤ 0.0001) or any advanced therapy (19 [9%] vs. 44 [19%]), primarily amongst patients with submassive PE [Table 2].

A landmark study in the treatment of acute PE was a randomized, double-blind trial (Fibrinolysis for Patients with Intermediate-Risk Pulmonary Embolism, PEITHO) by Meyer et al., in 2014, published in the *New England Journal of Medicine*, which assessed fibrinolysis for patients with an intermediate-risk PE [54] [Table 3], While this study did not evaluate PERT programs, its results influenced treatment algorithms for PERTs across the United States. In this study, there was a lower risk of death or hemodynamic decompensation in the fibrinolysis group with tenecteplase and heparin (13/506, 2.6%) versus in the placebo group which was treated with heparin alone (28/499, 5.6%) (odds ratio (OR) 0.44, 95% confidence interval (CI) 0.23–0.87, *p* = 0.02); however, the fibrinolytic group had a higher risk of bleeding (extracranial bleeding, stroke, etc.). Extracranial bleeding occurred in 6.3% of patients in the tenecteplase group compared to 1.2% in the placebo group (*p* < 0.001). Stroke occurred in 12 patients (2.4%) in the tenecteplase group (10 of which were hemorrhagic), while only 1 patient (0.2%) in the placebo group had a stroke (which was hemorrhagic, *p* = 0.003). By day 30, the mortality rate was 2.4% in the tenecteplase group and 3.2% in the placebo group (*p* = 0.42).

Among the numerous retrospective studies comparing pre-PERT and post-PERT metrics, the data are mixed [Table 2]. Some studies report a decrease in mortality following the formation of a PERT, while others find no significant difference. For instance, one study documented a decrease in in-hospital mortality from 16.5% pre-PERT to 9.5% post-PERT (*p* = 0.025), a median decrease of 3 days in total length of stay (LOS) (*p* < 0.001), and a reduction in ICU LOS by a median of 1.5 days (*p* = 0.023) [38]. Another study comparing outcomes for pre-PERT (n = 884) and post-PERT (n = 1158) patients presenting with a PE found no difference in PE-related mortality regardless of PERT activation (2.6% vs. 2.9%, *p* = 0.89); however, they observed a decrease in the use of systemic thrombolysis (3.8% vs. 2.1%, *p* = 0.02), a decrease in the placement of IVC filters (10.7% vs. 6.9%, *p* = 0.002), and an increase in catheter-directed therapies (1.3% vs. 3.3%, *p* = 0.05) [36]. A similar prospective review of patients with an imaging-confirmed PE in 2019 noted that of 307 patients, a PERT was activated for 22.5% and that advanced therapy use was significantly higher in the PERT cohort (35% vs. 2%), with no difference in major bleeds when comparing PERT activation against standard care [40]. This is in contrast to a retrospective review of 201 patients with PERT activations at a major teaching hospital in New York City, which noted that 16 patients (8.7%) had a PERT activated because of massive PEs but that the majority of patients were treated without invasive intervention (91.4% 95% CI 87.1–95.7%) [34]. 

In a separate study of 769 inpatients with a PE, researchers found that post-PERT patients had lower rates of major or clinically relevant nonmajor bleeding (17.0% vs. 8.3%, *p* = 0.002), a shorter time to therapeutic anticoagulation (16.3 h vs. 12.6 h, *p* = 0.009), and decreased use of inferior vena cava filters (22.2% vs. 16.4%, *p* = 0.004). Although an increase in the use of catheter-based therapy was noted, it was not statistically significant. Notably, there was a significant decrease in 30-day mortality (8.5% vs. 4.7%, *p* = 0.03), which was even more pronounced among intermediate and high-risk patients (10.0% vs. 5.3%, *p* = 0.02) [31]. 

When assessing PERT activation during the COVID-19 pandemic in New York City, one academic center, which had 82 activations between 1 March and 30 April 2020, noted that there were more PEs during the pandemic but fewer PERT activations (26.8% vs. 64.4%, *p* < 0.001) despite similar case characteristics [55]. They further noted that patients with PERT activations were more likely to be female, have a history of DVT, or a negative COVID polymerase-chain reaction test. This is in line with COVID-19-era recommendations that suggested careful patient selection for mechanical and surgical therapies to reduce facility contamination [19]. 

A smaller study at a tertiary-care center in Philadelphia analyzed 52 PERT activations postimplementation. In their cohort, 57.7% of cases were treated with anticoagulation alone, while 30.8% underwent catheter-directed thrombolysis. Although patients who underwent catheter-directed thrombolysis had a shorter intensive-care unit LOS, the difference was not statistically significant, and similar bleeding rates of approximately 6% were observed in both groups [32]. Another study from a tertiary-care hospital in Minnesota compared the following three periods of PE presentations: historical model, postconsensus-based treatment algorithm implementation, and post-PERT. There was a notable increase in the use of reperfusion therapy, both systemic thrombolysis and catheter-directed therapies, from 4.7% at baseline to 8.2% with the treatment algorithm and then to 16.1% with PERTs. This study also reported a statistically significant decrease in the length of stay by nearly 2 days [37]. Researchers at the University of Rochester Medical Center conducted an observational analysis of 137 pre-PERT- and 231 post-PERT-era patients with intermediate/high-risk PEs, observing a reduced 6-month mortality (14% vs. 25%, *p* = 0.025) and decreased LOS (6.5 days vs. 9.1 days, *p* = 0.007), and they found that an earlier diagnosis significantly impacted survival rates, with the risk of mortality decreasing by 5% for each hour earlier the diagnosis was made [41]. A single-center experience published at Cleveland Clinic noted that of 134 PERT activations between 2014 and 2016, PE was confirmed by imaging in 118 cases (88%), 14 patients (12%) were treated with catheter-directed therapies, 6 (5%) received full-dose systemic thrombolysis, 16 (13%) received half-dose systemic thrombolysis, and 10 (7%) underwent either surgical or mechanical thrombectomy. They noted zero bleeding events in patients regardless of systemic thrombolysis dosage [29]. Another study analyzed 279 patients who presented with PE, with a PERT activated for 133 (47.6%) of these cases. This study found reductions in both in-hospital (2% vs. 5%; *p* = 0.2) and 30-day mortality (2% vs. 8%; *p* = 0.06), although the 12-month mortality rates were similar between the groups (7% vs. 8%; *p* = 0.7) [56]. 

These studies offer insights into the benefits observed following the implementation of PERT programs across the United States, with similar findings reported internationally. In Spain, one small study involving 78 PERT activations compared to historically matched controls noted a significant reduction in 12-month mortality (9% vs. 22.2%, *p* = 0.02) [57]. Additionally, a study in Singapore reported decreased LOS and increased use of reperfusion therapy, without a corresponding increase in significant bleeding or changes in survival rates at discharge [58].

A recent meta-analysis found no difference in mortality when comparing the pre-PERT (357/3319, 10.8%) and post-PERT eras (265/3408, 7.8%) [risk ratio (RR) 0.89, 95% CI 0.67–1.19]. However, for patients specifically classified as intermediate or high risk, the mortality rate tended to be lower in the PERT era (142/1327, 10.7%) compared to the pre-PERT era (131/875, 15.0%) (RR 0.71, 95% CI 0.45–1.12). Additionally, the use of advanced therapies was higher (RR 2.67, 95% CI 1.29–5.50) and the in-hospital LOS was shorter by an average of 1.6 days [59]. The use of systemic thrombolysis and advanced therapies is an advantage of multidisciplinary PERTs especially considering that the International Cooperative of Pulmonary Embolism Registry and prior reports have noted as many as 70% of patients with a massive PE do not receive thrombolysis [60]. As more institutions join the PERT Consortium, the accumulation of data and the expansion of patient studies will enhance our understanding not only of how PERT is improving outcomes but also of which advanced therapies experts and evidence-based practices specifically recommend.

A retrospective review by Ardeshna et al. evaluated 817 patients (168 pre-PERT and 649 post-PERT) and noted that there was a decrease in advance therapy use (16% vs. 7.5%, *p* = 0.006), specifically catheter-based therapies (8.5% vs. 2.2%, *p* = 0.008) and IVC filter placement (5.3% vs. 3.2%, *p* < 0.001) in the post-PERT group [42]. A meta-analysis by Hobohm et al. included 22 original studies and 4 surveys with a total of 1532 intermediate-risk and high-risk patients evaluated by PERTs, and they noted that the overall mortality rate was 10%; however, when analyzing 9 controlled studies, there was no difference in mortality risk (risk ratio 0.89 95% CI 0.67–1.19) when comparing pre-PERT- and post-PERT-era patients [59]. These data are in contrast to prior presented reports and support the notion that PERT programs vary across centers while adhering to national guidelines. An etiology of these differences may stem from variations in the structures and responsibilities of PERTs across centers, including which specialists are available at local hospitals and other resource considerations [61].

From the perspective of emergency-room settings, a retrospective analysis of 425 patients treated by PERT programs over a 4-year time period noted that there was a decrease in primary endpoints (all-cause death, major bleeding, and venous thromboembolism) at 30 days (16.3% in 2015 vs. 7.1% in 2019), which persisted to 6 months (primary event rate 0.37, 95% CI 0.19–0.71) [43]. When comparing PERT activations by origin (emergency room vs. critical care unit vs. inpatient floors), one study found that activations from the ED (n = 283, 88/4%) or floor (n = 100, 74.6%) were more likely to be for confirmed PEs than activations from the ICU (n = 63, 58.9%; *p* < 0.0001) [44]. The ED was noted to have more PERT activations at night. Notably, the most PERT activations for massive PEs originated from a critical care setting (n = 41, 65.1%). Patient characteristics that have been previously studied and associated with PERT activation include vascular disease, pulmonary disease, central PE, and concurrent DVT, among other conditions, with one study commenting that only 15% (56/374) of low-risk PE patients triggered a PERT activation [45]. An opportunity to improve appropriate PERT activation lies in education and awareness. This has been documented in a prior study that noted education regarding PERT implementation programs have led to improved rates of providers identifying markers of high-risk PE and increased comfort in handling such cases [47].

Current advanced therapies for pulmonary embolism include systemic thrombolytic treatments with recombinant tissue plasminogen activators (tPAs), such as alteplase and tenecteplase, alongside newer device-based therapies. Although device-based therapies are relatively recent and have limited randomized controlled trial evidence supporting their use, their adoption has significantly increased following the implementation of PERTs, as demonstrated by the studies mentioned above.

Among the devices used for catheter-directed thrombolysis, the EkoSonic Endovascular System, or EKOS (Boston Scientific, Marlborough, MA, USA), was the first to gain FDA approval. This ultrasound-assisted catheter delivers thrombolysis directly at the PE site and is supported by a few randomized trials comparing it to anticoagulation or standard catheter-directed thrombolysis [46,62,63,64]. The Bashir Endovascular Catheter (Thrombolex) also delivers local thrombolysis through multiple ports and its efficacy is supported by several trials [48,65].

There are also devices which have been designed specifically for catheter-directed thrombectomy. The FlowTriever system (Inari Medical, Irvine, CA, USA), a syringe-based suction catheter, is used to retrieve clots and is supported by two nonrandomized trials [66,67]. The Indigo Aspiration System (Penumbra, Inc., Alameda, CA, USA), a continuous aspiration catheter system, has been validated in a clinical trial, demonstrating its efficacy [68]. Another FDA-approved device, the AngioVac (AngioDynamics, Latham, NY, USA), is a closed venous–venous system that uses extracorporeal circulatory support for PE extraction. AngioDynamics has also released the AlphaVac, which operates similarly but without the use of return cannulas. These devices provide PERT programs worldwide with additional options that can expedite and enhance treatment outcomes for pulmonary embolism.

The benefits of PERT are multifold and are linked to efficiency in the emergency department, decrease in utilization of resources, improved collaboration across services (emergency medicine, radiology, vascular surgery, interventional cardiology, interventional radiology, cardiothoracic surgery, vascular medicine, critical care, and hematology, among other specialties) and improved mortality benefit [69]. The main downside of implementing PERT programs is the cost to develop the program, increased administrative burden, and complexity. Initiating PERT programs requires coordination across medical specialties with the creation of an interdisciplinary team with a new call system to ensure 24/7 access to PERT capabilities [70]. 

## 4. Future Directions

The limitations of PERT programs are primarily linked to their relative novelty [33]. As PERT programs have developed extensively over the past decade, insufficient pre-PERT data exist to compare against current treatment outcomes. Similarly, randomized trials have also been limited because of the lack of an adequate patient cohort. Another limitation of PERT programs, as previously mentioned, is the increased administrative burden, which can lead to a more arduous multidisciplinary process to finalize treatment plans and potentially lead to delayed care. Further, many PERT programs are currently located in tertiary-care centers, which frequently offer invasive treatment courses that may otherwise not be as accessible at community sites, skewing PERT treatment data. The data are also evolving regarding the treatment of “low-risk” PE, which is a group of patients for whom guidelines are not as well developed [50]. Finally, insufficient long-term data exist on PERT management to determine the longitudinal impact of surgical vs. catheter-assisted therapies vs. fibrinolysis over time. 

As of 2024, there are over 100 medical centers that have joined the National PERT Consortium, which has led to the creation of a comprehensive network of physician leaders across specialties to tackle the problem of acute PE care. There are currently three randomized trials sponsored by the National PERT Consortium, which have previously been described [52], as follows: (1) APEX-AV, (2) HI-PEITHO, and (3) STORM-PE. 

The evidence is limited regarding direct head-to-head comparisons of treatments for patients with a moderate- or high-risk pulmonary embolism (PE), for which anticoagulation alone may be insufficient, and some form of advanced therapy is necessary. The PEERLESS clinical trial, which was recently initiated and is currently enrolling patients, addresses this gap. It compares large-bore mechanical thrombectomy using the FlowTriever System (Inari Medical, Irvine, CA, USA) with catheter-directed thrombolysis (CDT). Patients are randomized in a 1:1 ratio, and the outcomes include all-cause mortality, bleeding, clinical deterioration, and length of hospital stay. This trial will provide valuable evidence to guide the management of complex PEs once a PERT has decided to escalate treatment [49]. A similar trial that evolved from PEERLESS, the PEERLESS 2 trial (RCT of FlowTriever vs. anticoagulation alone in pulmonary embolism) is currently enrolling patients to understand treatment outcomes in intermediate-risk PEs [53]. 

As we look to the future, PERT programs will become commonplace in medical centers around the world, offering patients presenting with an acute PE the opportunity for standardized medical care regardless of the center.

## 5. Conclusions

Pulmonary embolism response teams (PERTs) have emerged as a critical innovation in the management of acute pulmonary embolism (PE), particularly for complex and high-risk cases. Since their inception in 2012, PERT programs have demonstrated significant benefits, including increased utilization of advanced therapies, reduced treatment variability, and improved patient outcomes. The multidisciplinary nature of PERTs ensures comprehensive, real-time decision making, which is crucial for optimizing treatment strategies and reducing mortality rates. Despite some limitations, such as the need for more randomized trials and long-term data, the positive impact of PERTs are evident. As PERT programs continue to expand globally and more institutions join the National PERT Consortium (https://pertconsortium.org/ accessed on 4 July 2024), the future of PE management looks promising, with ongoing research and collaboration set to further refine and enhance treatment protocols.

## Data Availability

No new data were created or analyzed in this study. Data sharing is not applicable to this article.

## References

[1] Barco S., Valerio L., Gallo A., Turatti G., Mahmoudpour S.H., Ageno W., Castellucci L.A., Cesarman-Maus G., Ddungu H., De Paula E.V. (2021). Global reporting of pulmonary embolism-related deaths in the World Health Organization mortality database: Vital registration data from 123 countries. Res. Pract. Thromb. Haemost..

[2] Eckelt J., Hobohm L., Merten M.C., Pagel C.F., Eggers A.-S., Lerchbaumer M.H., Stangl K., Hasenfuß G., Konstantinides S., Schmidtmann I. (2023). Long-term mortality in patients with pulmonary embolism: Results in a single-center registry. Res. Pract. Thromb. Haemost..

[3] Gupta R., Ammari Z., Dasa O., Ruzieh M., Burlen J.J., Shunnar K.M., Nguyen H.T., Xie Y., Brewster P., Chen T. (2020). Long-term mortality after massive, submassive, and low-risk pulmonary embolism. Vasc. Med..

[4] Stein P.D., Matta F. (2011). Epidemiology and incidence: The scope of the problem and risk factors for development of venous thromboembolism. Crit. Care Clin..

[5] Deitelzweig S.B., Johnson B.H., Lin J., Schulman K.L. (2011). Prevalence of clinical venous thromboembolism in the USA: Current trends and future projections. Am. J. Hematol..

[6] Aujesky D., Obrosky D.S., Stone R.A., Auble T.E., Perrier A., Cornuz J., Roy P.-M., Fine M.J. (2005). Derivation and validation of a prognostic model for pulmonary embolism. Am. J. Respir. Crit. Care Med..

[7] Russell C., Keshavamurthy S., Saha S. (2022). Classification and Stratification of Pulmonary Embolisms. Int. J. Angiol..

[8] Rivera-Lebron B., McDaniel M., Ahrar K., Alrifai A., Dudzinski D.M., Fanola C., Blais D., Janicke D., Melamed R., Mohrien K. (2019). Diagnosis, Treatment and Follow Up of Acute Pulmonary Embolism: Consensus Practice from the PERT Consortium. Clin. Appl. Thromb. Hemost..

[9] Jaff M.R., McMurtry M.S., Archer S.L., Cushman M., Goldenberg N., Goldhaber S.Z., Jenkins J.S., Kline J.A., Michaels A.D., Thistlethwaite P. (2011). Management of massive and submassive pulmonary embolism, iliofemoral deep vein thrombosis, and chronic thromboembolic pulmonary hypertension: A scientific statement from the American Heart Association. Circulation.

[10] Stevens S.M., Woller S.C., Kreuziger L.B., Bounameaux H., Doerschug K., Geersing G.J., Huisman M.V., Kearon C., King C.S., Knighton A.J. (2021). Antithrombotic Therapy for VTE Disease: Second Update of the CHEST Guideline and Expert Panel Report. Chest.

[11] Ortel T.L., Neumann I., Ageno W., Beyth R., Clark N.P., Cuker A., Hutten B.A., Jaff M.R., Manja V., Schulman S. (2020). American Society of Hematology 2020 guidelines for management of venous thromboembolism: Treatment of deep vein thrombosis and pulmonary embolism. Blood Adv..

[12] Konstantinides S.V., Meyer G., Becattini C., Bueno H., Geersing G.J., Harjola V.P., Huisman M.V., Humbert M., Jennings C.S., Jiménez D. (2020). 2019 ESC Guidelines for the diagnosis and management of acute pulmonary embolism developed in collaboration with the European Respiratory Society (ERS). Eur. Heart J..

[13] Goldhaber S.Z., Visani L., De Rosa M. (1999). Acute pulmonary embolism: Clinical outcomes in the International Cooperative Pulmonary Embolism Registry (ICOPER). Lancet.

[14] Monteleone P.P., Rosenfield K., Rosovsky R.P. (2016). Multidisciplinary pulmonary embolism response teams and systems. Cardiovasc. Diagn. Ther..

[15] Rosovsky R., Borges J., Kabrhel C., Rosenfield K. (2018). Pulmonary Embolism Response Team: Inpatient Structure, Outpatient Follow-up, and Is It the Current Standard of Care?. Clin. Chest Med..

[16] Barnes G., Giri J., Courtney D.M., Naydenov S., Wood T., Rosovsky R., Rosenfield K., Kabrhel C. (2017). Nuts and bolts of running a pulmonary embolism response team: Results from an organizational survey of the National PERT™ Consortium members. Hosp. Pract..

[17] Rosovsky R., Chang Y., Rosenfield K., Channick R., Jaff M.R., Weinberg I., Sundt T., Witkin A., Rodriguez-Lopez J., Parry B.A. (2019). Changes in treatment and outcomes after creation of a pulmonary embolism response team (PERT), a 10-year analysis. J. Thromb. Thrombolysis.

[18] Rosovsky R.P., Grodzin C., Channick R., Davis G.A., Giri J.S., Horowitz J., Kabrhel C., Lookstein R., Merli G., Morris T.A. (2020). Diagnosis and Treatment of Pulmonary Embolism during the Coronavirus Disease 2019 Pandemic: A Position Paper from the National PERT Consortium. Chest.

[19] Finn M.T., Gogia S., Ingrassia J.J., Cohen M., Madhavan M.V., Nouri S.N., Brailovsky Y., Masoumi A., Fried J.A., Uriel N. (2021). Pulmonary Embolism Response Team utilization during the COVID-19 pandemic. Vasc. Med..

[20] Hobohm L., Farmakis I.T., Duerschmied D., Keller K. (2024). The Current Evidence of Pulmonary Embolism Response Teams and Their Role in Future. Hamostaseologie.

[21] Myc L.A., Solanki J.N., Barros A.J., Nuradin N., Nevulis M.G., Earasi K., Richardson E.D., Tsutsui S.C., Enfield K.B., Teman N.R. (2020). Adoption of a dedicated multidisciplinary team is associated with improved survival in acute pulmonary embolism. Respir. Res..

[22] Rali P., Sacher D., Rivera-Lebron B., Rosovsky R., Elwing J.M., Berkowitz J., Mina B., Dalal B., Davis G.A., Dudzinski D.M. (2021). Interhospital Transfer of Patients with Acute Pulmonary Embolism: Challenges and Opportunities. Chest.

[23] Rivera-Lebron B.N., Rali P.M., Tapson V.F. (2021). The PERT Concept: A Step-by-Step Approach to Managing Pulmonary Embolism. Chest.

[24] Dudzinski D.M., Piazza G. (2016). Multidisciplinary Pulmonary Embolism Response Teams. Circulation.

[25] Mathis G., Blank W., Reissig A., Lechleitner P., Reuß J., Schuler A., Beckh S. (2005). Thoracic ultrasound for diagnosing pulmonary embolism: A prospective multicenter study of 352 patients. Chest.

[26] Hobohm L., Sagoschen I., Habertheuer A., Barco S., Valerio L., Wild J., Schmidt F.P., Gori T., Münzel T., Konstantinides S. (2022). Clinical use and outcome of extracorporeal membrane oxygenation in patients with pulmonary embolism. Resuscitation.

[27] Keeling W.B., Sundt T., Leacche M., Okita Y., Binongo J., Lasajanak Y., Aklog L., Lattouf O.M. (2016). Outcomes After Surgical Pulmonary Embolectomy for Acute Pulmonary Embolus: A Multi-Institutional Study. Ann. Thorac. Surg..

[28] Maqsood M.H., Zhang R., Zlotnick D., Parikh S., Bangalore S. (2024). Outcomes with treatment interventions for clot-in-transit in patients with pulmonary embolism: A meta-analysis. J. Invasive Cardiol..

[29] Mahar J.H., Haddadin I., Sadana D., Gadre A., Evans N., Hornacek D., Mahlay N.F., Gomes M., Joseph D., Serhal M. (2018). A pulmonary embolism response team (PERT) approach: Initial experience from the Cleveland Clinic. J. Thromb. Thrombolysis.

[30] Sista A.K., Friedman O.A., Dou E., Denvir B., Askin G., Stern J., Estes J., Salemi A., Winokur R.S., Horowitz J.M. (2018). A pulmonary embolism response team’s initial 20 month experience treating 87 patients with submassive and massive pulmonary embolism. Vasc. Med..

[31] Chaudhury P., Gadre S.K., Schneider E., Renapurkar R.D., Gomes M., Haddadin I., Heresi G.A., Tong M.Z., Bartholomew J.R. (2019). Impact of Multidisciplinary Pulmonary Embolism Response Team Availability on Management and Outcomes. Am. J. Cardiol..

[32] Khaing P., Paruchuri A., Eisenbrey J.R., Merli G.J., Gonsalves C.F., West F.M., Awsare B.K. (2020). First year experience of a pulmonary embolism response team with comparisons of outcomes between catheter directed therapy versus standard anticoagulation. Hosp. Pract..

[33] Schultz J., Giordano N., Zheng H., Parry P.A., Barnes G.D., Heresi G.A., Jaber W., Wood T., Todoran T., Courtney D.M. (2019). EXPRESS: A Multidisciplinary Pulmonary Embolism Response Team (PERT)—Experience from a national multicenter consortium. Pulm. Circ..

[34] Wiske C.P., Shen C., Amoroso N., Brosnahan S.B., Goldenberg R., Horowitz J., Jamin C., Sista A.K., Smith D., Maldonado T.S. (2020). Evaluating time to treatment and in-hospital outcomes of pulmonary embolism response teams. J. Vasc. Surg. Venous Lymphat. Disord..

[35] Xenos E.S., Davis G.A., He Q., Green A., Smyth S.S. (2019). The implementation of a pulmonary embolism response team in the management of intermediate- or high-risk pulmonary embolism. J. Vasc. Surg. Venous Lymphat. Disord..

[36] Carroll B.J., Beyer S.E., Mehegan T., Dicks A., Pribish A., Locke A., Godishala A., Soriano K., Kanduri J., Sack K. (2020). Changes in Care for Acute Pulmonary Embolism through a Multidisciplinary Pulmonary Embolism Response Team. Am. J. Med..

[37] Melamed R., Hill C.A.S., Engstrom B.I., Tierney D.M., Smith C.S., Agboto V.K., Weise B.E., Eckman P.M., Skeik N. (2020). Effects of a Consensus-Based Pulmonary Embolism Treatment Algorithm and Response Team on Treatment Modality Choices, Outcomes, and Complications. Clin. Appl. Thromb. Hemost..

[38] Annabathula R., Dugan A., Bhalla V., Davis G.A., Smyth S.S., Gupta V.A. (2021). Value-based assessment of implementing a Pulmonary Embolism Response Team (PERT). J. Thromb. Thrombolysis.

[39] Araszkiewicz A., Kurzyna M., Kopeć G., Sławek-Szmyt S., Wrona K., Stępniewski J., Jankiewicz S., Pietrasik A., Machowski M., Darocha S. (2021). Pulmonary embolism response team: A multidisciplinary approach to pulmonary embolism treatment. Polish PERT Initiative Report. Kardiol. Pol..

[40] Parikh M., Chahine N.M., Hammad T.A., Tefera L., Li J., Carman T., Schilz R., Shishehbor M.H. (2021). Predictors and potential advantages of PERT and advanced therapy use in acute pulmonary embolism. Catheter. Cardiovasc. Interv..

[41] Wright C., Goldenberg I., Schleede S., McNitt S., Gosev I., Elbadawi A., Pietropaoli A., Barrus B., Chen Y.L., Mazzillo J. (2021). Effect of a Multidisciplinary Pulmonary Embolism Response Team on Patient Mortality. Am. J. Cardiol..

[42] Ardeshna N.S., Song M., Hyder S.N., Grace K.A., O’Hare C., Schaeffer W.J., Stover M., Greineder C.F., Barnes G.D. (2023). Effect of pulmonary embolism response team on advanced therapies administered: The University of Michigan experience. Thromb. Res..

[43] Chopard R., Campia U., Morin L., Jering K.S., Almarzooq Z.I., Snyder J.E., Rizzo S., Waxman A.B., Goldhaber S.Z., Piazza G. (2022). Trends in management and outcomes of pulmonary embolism with a multidisciplinary response team. J. Thromb. Thrombolysis.

[44] Deadmon E.K., Giordano N.J., Rosenfield K., Rosovsky R., Parry B.A., Al-Bawardy R.F., Chang Y., Kabrhel C. (2017). Comparison of Emergency Department Patients to Inpatients Receiving a Pulmonary Embolism Response Team (PERT) Activation. Acad. Emerg. Med..

[45] Mortensen C.S., Kramer A., Schultz J.G., Giordano N., Zheng H., Andersen A., Nielsen-Kudsk J.E., Kabrhel C. (2022). Predicting factors for pulmonary embolism response team activation in a general pulmonary embolism population. J. Thromb. Thrombolysis.

[46] Tapson V.F., Sterling K., Jones N., Elder M., Tripathy U., Brower J., Maholic R.L., Ross C.B., Natarajan K., Fong P. (2018). A Randomized Trial of the Optimum Duration of Acoustic Pulse Thrombolysis Procedure in Acute Intermediate-Risk Pulmonary Embolism: The OPTALYSE PE Trial. JACC Cardiovasc. Interv..

[47] Brailovsky Y., Kunchakarra S., Lakhter V., Barnes G., Masic D., Mancl E., Porcaro K., Bechara C.F., Lopez J.J., Simpson K. (2020). Pulmonary embolism response team implementation improves awareness and education among the house staff and faculty. J. Thromb. Thrombolysis.

[48] Sista A.K., Bhatheja R., Rali P., Natarajan K., Green P., Piazza G., Comerota A.J., Parikh S.A., Lakhter V., Bashir R. (2021). First-in-Human Study to Assess the Safety and Feasibility of the Bashir Endovascular Catheter for the Treatment of Acute Intermediate-Risk Pulmonary Embolism. Circ. Cardiovasc. Interv..

[49] Gonsalves C.F., Gibson C.M., Stortecky S., Alvarez R.A., Beam D.M., Horowitz J.M., Silver M.J., Toma C., Rundback J.H., Rosenberg S.P. (2023). Randomized controlled trial of mechanical thrombectomy vs catheter-directed thrombolysis for acute hemodynamically stable pulmonary embolism: Rationale and design of the PEERLESS study. Am. Heart J..

[50] Fleitas Sosa D., Lehr A.L., Zhao H., Roth S., Lakhther V., Bashir R., Cohen G., Panaro J., Maldonado T.S., Horowitz J. (2022). Impact of pulmonary embolism response teams on acute pulmonary embolism: A systematic review and meta-analysis. Eur. Respir. Rev..

[51] Silver M.J., Gibson C.M., Giri J., Khandhar S., Jaber W., Toma C., Mina B., Bowers T., Greenspon L., Kado H. (2023). Outcomes in High-Risk Pulmonary Embolism Patients Undergoing FlowTriever Mechanical Thrombectomy or Other Contemporary Therapies: Results from the FLAME Study. Circ. Cardiovasc. Interv..

[52] Consortium P. (2024). PERT Consortium. https://pertconsortium.org/.

[53] Giri J., Mahfoud F., Gebauer B., Andersen A., Friedman O., Gandhi R.T., Jaber W.A., Pereira K., West F.M. (2024). PEERLESS II: A Randomized Controlled Trial of Large-Bore Thrombectomy versus Anticoagulation in Intermediate-Risk Pulmonary Embolism. J. Soc. Cardiovasc. Angiogr. Interv..

[54] Meyer G., Vicaut E., Danays T., Agnelli G., Becattini C., Beyer-Westendorf J., Bluhmki E., Bouvaist H., Brenner B., Couturaud F. (2014). Fibrinolysis for patients with intermediate-risk pulmonary embolism. N. Engl. J. Med..

[55] Kwok B., Brosnahan S.B., Amoroso N.E., Goldenberg R.M., Heyman B., Horowitz J.M., Jamin C., Sista A.K., Smith D.E., Yuriditsky E. (2021). Pulmonary Embolism Response Team activation during the COVID-19 pandemic in a New York City Academic Hospital: A retrospective cohort analysis. J. Thromb. Thrombolysis.

[56] Russell N., Sayfo S., George T., Gable D. (2023). Effect of a pulmonary embolism response team on the management and outcomes of patients with acute pulmonary embolism. J. Vasc. Surg. Venous Lymphat. Disord..

[57] Ramos-López N., Ferrera C., Luque T., Enríquez-Vázquez D., Mahía-Casado P., Galván-Herráez L., Pedrajas J.M., Salinas P. (2023). Impact of a pulmonary embolism response team initiative on hospital mortality of patients with bilateral pulmonary embolism. Med. Clin..

[58] Jen W.-Y., Kristanto W., Teo L., Phua J., Yip H.S., MacLaren G., Teoh K., Sim T.B., Loh J., Ong C.C. (2020). Assessing the Impact of a Pulmonary Embolism Response Team and Treatment Protocol on Patients Presenting with Acute Pulmonary Embolism. Heart Lung Circ..

[59] Hobohm L., Farmakis I.T., Keller K., Scibior B., Mavromanoli A.C., Sagoschen I., Münzel T., Ahrens I., Konstantinides S. (2023). Pulmonary embolism response team (PERT) implementation and its clinical value across countries: A scoping review and meta-analysis. Clin. Res. Cardiol..

[60] Thangudu P. (2024). From Trendelenburg to PERTs: Evolution in the Management of Massive Pulmonary Embolism. Methodist Debakey Cardiovasc. J..

[61] Porres-Aguilar M., Rosovsky R.P., Rivera-Lebron B.N., Kaatz S., Mukherjee D., Anaya-Ayala J.E., Jimenez D., Jerjes-Sánchez C. (2022). Pulmonary embolism response teams: Changing the paradigm in the care for acute pulmonary embolism. J. Thromb. Haemost..

[62] Kucher N., Boekstegers P., Müller O.J., Kupatt C., Beyer-Westendorf J., Heitzer T., Tebbe U., Horstkotte J., Müller R., Blessing E. (2014). Randomized, controlled trial of ultrasound-assisted catheter-directed thrombolysis for acute intermediate-risk pulmonary embolism. Circulation.

[63] Piazza G., Hohlfelder B., Jaff M.R., Ouriel K., Engelhardt T.C., Sterling K.M., Jones N.J., Gurley J.C., Bhatheja R., Kennedy R.J. (2015). A Prospective, Single-Arm, Multicenter Trial of Ultrasound-Facilitated, Catheter-Directed, Low-Dose Fibrinolysis for Acute Massive and Submassive Pulmonary Embolism: The SEATTLE II Study. JACC Cardiovasc. Interv..

[64] Avgerinos E.D., Jaber W., Lacomis J., Markel K., McDaniel M., Rivera-Lebron B.N., Ross C.B., Sechrist J., Toma C., Chaer R. (2021). Randomized Trial Comparing Standard Versus Ultrasound-Assisted Thrombolysis for Submassive Pulmonary Embolism: The SUNSET sPE Trial. JACC Cardiovasc. Interv..

[65] Bashir R., Foster M., Iskander A., Darki A., Jaber W., Rali P.M., Lakhter V., Gandhi R., Klein A., Bhatheja R. (2022). Pharmacomechanical Catheter-Directed Thrombolysis with the Bashir Endovascular Catheter for Acute Pulmonary Embolism: The RESCUE Study. JACC Cardiovasc. Interv..

[66] Tu T., Toma C., Tapson V.F., Adams C., Jaber W.A., Silver M., Khandhar S., Amin R., Weinberg M., Engelhardt T. (2019). A Prospective, Single-Arm, Multicenter Trial of Catheter-Directed Mechanical Thrombectomy for Intermediate-Risk Acute Pulmonary Embolism: The FLARE Study. JACC Cardiovasc. Interv..

[67] Toma C., Bunte M.C., Cho K.H., Jaber W.A., Chambers J., Stegman B., Gondi S., Leung D.A., Khandhar S., Kado H. (2022). Percutaneous mechanical thrombectomy in a real-world pulmonary embolism population: Interim results of the FLASH registry. Catheter. Cardiovasc. Interv..

[68] Sista A.K., Horowitz J.M., Tapson V.F., Rosenberg M., Elder M.D., Schiro B.J., Dohad S., Amoroso N.E., Dexter D.J., Loh C.T. (2021). Indigo Aspiration System for Treatment of Pulmonary Embolism: Results of the EXTRACT-PE Trial. JACC Cardiovasc. Interv..

[69] Bejjani A., Khairani C.D., Campia U., Piazza G. (2022). Pulmonary Embolism Response Teams: Theory, Implementation, and Unanswered Questions. J. Clin. Med..

[70] Brailovsky Y., Lakhter V. (2021). Pulmonary Embolism Response Team: Additional Call Burden or a Valuable Learning Opportunity?. J. Am. Coll. Cardiol..

